# Effect of an *Escherichia coli*–derived phytase and a carbohydrase–protease cocktail derived from *Bacillus* spp. on performance, digestibility, bone mineralization and gut morphology in broilers fed different nutrient density diets

**DOI:** 10.1002/vms3.1344

**Published:** 2024-01-16

**Authors:** Mostafa Ahmadi, Hossein Ali Ghasemi, Iman Hajkhodadadi, Farhad Khaligh

**Affiliations:** ^1^ Department of Animal Science Faculty of Agriculture and Environment Arak University Arak Iran; ^2^ Department of Animal Science Faculty of Agriculture Ferdowsi University of Mashhad Mashhad Iran

**Keywords:** bone quality, broilers, dietary nutrient, intestinal health, multi‐enzyme, phytase

## Abstract

**Background:**

Enzyme combinations, particularly phytase (PHY) with various carbohydrases and proteases, are utilized in commercial broiler production to enhance nutrient and energy bioavailability.

**Objective:**

A feeding study was undertaken to determine whether the efficiency of an *Escherichia coli*–derived PHY and a feed enzyme complex (FEC) derived from *Bacillus* spp. containing carbohydrase and protease as main activities in broiler chickens is dependent on diet quality. A total of 900 male one‐day‐old broiler chickens (Ross 308) were assigned to a 2 × 3 factorial arrangement of the treatments with 2 different nutrient density diets, standard nutrient diet (SN diet) and a low‐nutrient diet (LN diet; −100 kcal/kg for AMEn and −5% for crude protein [CP] and limiting amino acids), and 3 enzyme treatments (control [no enzymes], PHY and PHY + FEC). Each treatment group was composed of 6 replicates of 25 birds each.

**Results:**

The LN diet caused a decrease in performance index, tibia length and diameter, tibia calcium content and jejunal villus surface area (VSA). The interaction effects between diet and enzyme supplementation were observed (*p* < 0.05) on overall average daily gain (ADG), performance index, tibia ash content and jejunal villus height (VH) and VSA, with the favourable benefits of PHY + FEC treatment being more pronounced in the LN diets. Regardless of dietary nutrient density, supplementation with PHY alone or combined with FEC enhanced (*p* < 0.05) final body weight, overall ADG and jejunal villus height (VH)/crypt depth, with the highest values observed in the PHY + FEC group. The PHY + FEC treatment also improved (*p* < 0.05) overall feed conversion ratio, apparent ileal digestibility of dry matter, organic matter, CP, and energy, and tibia phosphorus content compared to the control treatment.

**Conclusions:**

The results indicate that the simultaneous addition of PHY and FEC to the LN diets improved the growth rate, bone mineralization and gut morphology.

## Graphical abstract

- Efficacy of phytase (PHY) and a feed enzyme complex (FEC) were evaluated in broilers fed two different diets.
- Feeding a low‐nutrient diet had an adverse effect on performance, bone mineralization and gut morphology.
- Supplementing with PHY + FEC improved growth rate and intestinal morphology in low‐nutrient diets.
- The benefits of PHY and FEC were not dependent on the quality of the diet in terms of nutrient digestibility or bone quality.

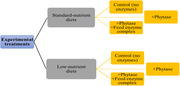

## 1 INTRODUCTION

Diets low in crude protein (CP) or metabolizable energy (ME) have gained considerable attention in poultry nutrition over the last few decades as a means of reducing feed costs and potential environmental challenges (Law et al., 2019). However, lowering CP and/or ME below a certain level may have a negative influence on feed intake and growth rate (Boontiam et al., 2017; Jlali et al., 2020). The inclusion of exogenous enzymes has proven to be an efficient cost‐saving approach. Exogenous enzymes can contribute to nutritional advantages in a variety of ways, one of which is by hydrolyzing non‐starch polysaccharides (NSPs) that the bird cannot utilize due to their structure (Gallardo et al., 2017; Lu et al., 2013). For some substances, this results in a reduction in intestinal viscosity as well as an increase in dietary energy expenditure by the bird (Bilal et al., 2017). On the other hand, poultry species are also unable to use phytate‐bound phosphorus in corn–soya bean–based diets because they lack an endogenous phytase (PHY) capable of hydrolyzing inositol–phosphate bonds (Akter et al., 2016; Liu & Ru, 2010). Despite the fact that gut viscosity is not typically a concern with corn–soya bean meal diets, the NSP compounds and phytate contained in these types of diets have the potential to encapsulate a variety of nutrients, thereby impairing their utilization (Aftab & Bedford, 2018; Babaei et al., 2021; Bedford & Rousseau, 2017). Supplementing broiler chicken diets with PHY‐ and NSP‐degrading enzymes (e.g. xylanase, β‐glucanase and pectinase) can offset the unfavourable effects of phytate and NSP on digestion, resulting in improved productivity.

PHY supplementation has been studied in several poultry species, including broilers (Attia et al., 2021; Dersjant‐Li et al., 2018), laying hens (Herwig et al., 2021; Mohebbifar et al., 2015) and quails (Sajadi Hezaveh et al., 2020). The results of these investigations have consistently shown that PHY supplementation enhances dietary phosphorous availability across all studies and species studied. In addition, multiple studies have shown that PHY increases the availability of other minerals (Akter et al., 2016; Mohammadi Ziarat et al., 2020) and amino acids (Jing et al., 2021; Jlali et al., 2020), which in turn enhances growth rate and bone mineralization. A recent study conducted on broiler chickens (Moss et al., 2020) has shown that PHY supplementation increased starch digestibility and energy utilization of maize‐ and wheat‐based diets, which was associated with an increase in NSP digestibility. According to a prior investigation conducted by Sharma et al. (2016), the utilization of nutrient matrix values in the formulation of PHY‐supplemented diets yields greater advantages compared to its addition to an already nutrient‐sufficient diet.

The findings from investigations on NSP‐degrading enzymes in broiler chickens are less conclusive than those from studies involving PHY. In some trials, no or just minimal impacts were detected (Karimi et al., 2013; Noormohammadi et al., 2023; Tiwari et al., 2010), but in others, greater nutrient digestibility and growth rate were discovered (Cozannet et al., 2019; Kiarie et al., 2021). It is difficult to compare the effects of NSP‐degrading enzymes across studies because of the range of types and doses of enzymes employed separately or in combination. However, changes in nutritional and/or component composition of diets may account for the majority of the variability, because the NSP content of the feed influences the response to exogenous enzymes in terms of productive performance and nutrient digestibility (Aftab & Bedford, 2018; Cowieson & Bedford, 2009). In contrast to the investigations that were conducted on PHY‐ and NSP‐degrading enzymes, protease has not been widely used in boiler diets. An improvement in the digestibility of dietary amino acids by the use of exogenous protease has been demonstrated to be economically advantageous for broiler production (Romero et al., 2013). According to Jabbar et al. (2021), the addition of protease to broiler diets can enhance feed efficiency and nutrient digestibility in broilers during the starter phase.

Based on the described findings, it is postulated that the response to enzyme supplementation can be affected by the levels of ME, CP and amino acids in the diets as well as by the amount of the target substrate consumed (e.g. phytate or NSP). In the current study, it is expected that the simultaneous addition of PHY and multi‐enzyme complex could enhance nutrient and energy utilization in broilers given low‐nutrient diets (LN diets) (low ME, CP and limiting amino acid [LAA] contents), allowing these birds to perform at a comparable level to birds fed a standard diet. Until now, no broiler studies have been conducted to examine whether the effect of PHY and multi‐enzyme complex is dependent on the nutrient density of the feed, that is the quantity of dietary energy and CP. Thus, the objective of this study was to examine if lowering the amount of ME, CP and LAA with and without the addition of an *Escherichia coli*–derived PHY and a feed enzyme complex (FEC) derived from *Bacillus* spp. containing carbohydrase and protease as main activities would affect growth performance, nutrient digestibility, bone characteristics and gut morphology in broiler chickens.

## 2 MATERIALS AND METHODS

### 2.1 Animal ethics

The authors attest that they have obtained approval from the Arak University Institutional Animal Care and Use Committee (contract number 1400/1911) and have followed the journal's ethical policies as outlined on the journal's author guidelines page. The authors have also complied with all regulations regarding the protection of animals used for scientific purposes.

### 2.2 Diets and experimental design

A total of 900 Ross 308 male broiler chickens (average weight = 43.4 ± 0.65 g, 1‐day old) were obtained from a local commercial hatchery and randomly assigned to 6 dietary treatment groups and 6 replicate pens (25 birds/pen with a size of 2 m × 1.75 m). The impacts of enzyme supplementation in broiler chickens were evaluated using a standard nutrient diet (SN diet) and an LN diet. These diets were supplemented with either PHY (SN + PHY, LN + PHY), PHY and FEC (SN + PHY + FEC, LN + PHY + FEC) or not supplemented (SN + 0, LN + 0). The SN diet was formulated to meet all major nutrient requirements as recommended by breeders, whereas the LN diet was designed to be nutritionally deficient in AMEn (−100 kcal/kg), CP and digestible amino acids (95% of the commercially recommended levels). The avian subjects were provided with a starter diet during the initial period of days 0–10, followed by a grower diet from days 10 to 24 and subsequently a finisher diet from days 24 to 42. The feed ingredients and chemical contents of the SN and LN diets are presented in Table 1. The experiment lasted 6 weeks (from days 0 to 42), during which the birds had unlimited access to feed (in mash form). The lighting scheme was 24 h/day for the first 7 days and then dropped to 23 h/day throughout the experiment. During the first week, the temperature was maintained at 32°C and gradually declined to 23°C at the end of the fourth week. During the experiment, the relative humidity was 55% ± 5%, which was achieved by employing a humidifier inside the room.

**TABLE 1:** Ingredient composition and nutrient contents of starter, grower and finisher diets (as fed basis).

	Starter (days 0–10)		Grower (days 10–24)		Finisher (days 24–42)	
Dietary AMEn and CP levels	Standard	Low	Standard	Low	Standard	Low
Ingredients (g/kg)						
Corn grain	564.8	582.9	599.6	614.0	648.5	663.5
Soybean meal	307.1	344.5	267.5	308.5	218.4	255.7
Corn gluten meal	60.0	15.0	60.9	15.0	57.1	15.0
Soya bean oil	18.6	10.5	27.0	19.8	34.0	25.8
Dicalcium phosphate	19.6	19.4	17.6	17.4	15.8	15.6
Limestone	11.6	11.4	10.6	10.4	9.8	9.7
Salt (NaCl)	2.2	2.5	1.9	2.4	1.8	2.2
Sodium bicarbonate	1.0	0.5	1.4	0.7	1.6	1.0
Vitamin premix^a^	2.5	2.5	2.5	2.5	2.5	2.5
Mineral premix^b^	2.5	2.5	2.5	2.5	2.5	2.5
l‐Lysine.HCL	5.1	3.4	4.5	2.8	4.4	2.9
dl‐Methionine	2.7	2.9	2.2	2.5	2.0	2.2
l‐Threonine	2.3	2.0	1.8	1.5	1.6	1.4
Calculated nutritive value (g/kg unless stated otherwise)						
Metabolizable energy, kcal/kg	3000	2900	3100	3000	3200	3100
Crude protein	230.0	218.5	215.0	204.2	195.0	185.2
Calcium	9.6	9.6	8.7	8.7	7.9	7.9
Available phosphorus	4.8	4.8	4.4	4.4	4.0	4.0
Digestible lysine	12.8	12.2	11.5	10.9	10.3	9.8
Digestible methionine	6.2	5.9	5.5	5.3	5.1	4.9
Digestible total sulphur amino acids	9.5	9.0	8.7	8.3	8.0	7.6
Digestible threonine	8.6	8.2	7.7	7.3	6.9	6.6
DEB^c^, mEq/100 g	245	250	230	236	220	230
Analysed nutritive value^d^ (g/kg)						
Gross energy, kcal/kg	4522	4443	4567	4491	4596	4517
Crude protein	226.4	215.1	212.5	200.6	190.6	181.0
Crude fat	43.9	35.8	53.4	45.9	61.8	53.5
Calcium	9.9	9.8	8.9	8.9	8.0	7.9
Total phosphorus	7.6	7.6	7.0	7.1	6.5	6.5
Total lysine	14.7	14.2	13.2	12.7	11.8	11.4
Total methionine	6.9	6.5	6.2	5.9	5.8	5.5
Total cystine	3.6	3.4	3.4	3.2	3.1	2.9
Total sulphur amino acids	10.5	9.9	9.7	9.2	8.9	8.4
Total threonine	9.9	9.5	8.5	8.0	7.7	7.3
Total valine	11.7	11.4	11.0	10.7	10.0	9.8
Total tryptophan	2.6	2.6	2.3	2.2	2.1	2.0
Total arginine	14.1	14.3	12.9	13.2	11.5	11.9
Total glycine	10.1	9.9	9.3	9.0	8.3	8.0
Total isoleucine	10.8	10.7	10.0	9.9	8.9	8.8

Abbreviation: CP, crude protein.

^a^Supplies per kg of the diet: vitamin A (retinyl acetate), 11,000 IU; vitamin D3 (cholecalciferol), 1800 IU; vitamin E (dl‐α‐tocopheryl acetate), 11 mg; vitamin K3 (menadione dimethylpyrimidinol), 2 mg; thiamin (thiamine mononitrate), 1.6 mg; riboflavin, 6 mg; niacin, 30 mg; d‐calcium pantothenate, 15 mg; pyridoxine, 2 mg; biotin, 0.25 mg; folic acid, 0.8 mg; vitamin B12, 0.020 mg; choline (choline chloride), 500 mg.

^b^Supplies per kg of the diet: Mn (manganese oxide), 60 mg; Zn (zinc sulphate), 60 mg; Fe (ferrous sulphate), 50 mg; Cu (cupric sulphate), 10 mg; I (potassium iodide), 1 mg; Se (sodium selenite), 0.30 mg.

^c^DEB (dietary electrolyte balance) = (Na^+^, mEq/kg + K^+^, mEq/kg) − CL^−^, mEq/kg.

^d^Mean of two samples per diet (Evonik Industries, Evonik Degussa GmbH).

### 2.3 Phytase and multi‐enzyme

The PHY used in this study, Microtech 10000 Plus (Guangdong VTR Bio‐Tech Co., Ltd.), was a granular bacterial 6‐PHY (*E. coli*–derived) expressed in *Pichia pastoris*. Each gram of Microtech 10000 Plus contains 45,000 to 60,000 particles with an overall minimum activity of 10,000 FTU. The enzyme was added to the feed at 100 mg (1000 FTU)/kg. The PHY activity of the PHY‐supplemented diet was determined using the technique used by Slominski et al. (2007).

A multi‐enzyme complex (MultiBehzyme, AKAM Faravardehaye Bahman Arad Co., Karaj, Iran) in powder form was tested, containing endo‐1,4‐β‐xylanase produced from *Bacillus subtilis* (EC 3.2.1.8), β‐glucanase produced from *Bacillus amyloliquefaciens* (EC 3.2.1.6), pectinase produced from *Bacillus licheniformis* (EC 3.2.1.15), α‐amylase produced from *B. amyloliquefaciens* (EC 3.2.1.1) and protease produced from *B. subtilis* (EC 3.4.21.62). The multi‐enzyme complex was administered at a dosage rate of 200 mg/kg of feed to ensure a minimum of 500 U amylase, 2000 U xylanase, 600 U pectinase, 800 U β‐glucanase and 3000 U protease per kg of feed. The proportion of enzymes recovered from feed was measured by the enzyme manufacturer (Table 2).

**TABLE 2:** Supplemental level and analysed activity^a^ of exogenous enzymes in experimental diets.

	Phytase	Xylanase	β‐Glucanase	Pectinase	Amylase	Protease
Item	FTU/kg	XU/kg	IU/kg	IU/kg	AU/kg	PCT/kg
Expected in all diets	1000	2000	800	600	500	3000
Analysed values						
Starter diets^b^						
SN + 0	<100	0	<30	0	<1	0
SN + PHY	1292	0	<30	0	<1	0
SN + PHY + FEC	1305	2321	762	542	588	2657
LN + 0	<100	0	<30	0	<1	0
LN + PHY	1261	0	<30	0	<1	0
LN + PHY + FEC	1287	2355	726	495	560	2845
Grower diets^b^						
SN + 0	<100	0	<30	0	<1	0
SN + PHY	1264	0	<30	0	<1	0
SN + PHY + FEC	1226	1956	736	542	583	3345
LN + 0	<100	0	<30	0	<1	0
LN + PHY	1308	0	<30	0	<1	0
LN + PHY + FEC	1323	2155	690	544	564	3245
Finisher diets^b^						
SN + 0	<100	0	<30	0	<1	0
SN + PHY	1322	0	<30	0	<1	0
SN + PHY + FEC	1278	2260	894	540	578	3605
LN + 0	<100	0	<30	0	<1	0
LN + PHY	1296	0	<30	0	<1	0
LN + PHY + FEC	1310	2341	881	552	535	3510

^a^One FTU was defined as the amount of enzyme necessary to release 1 mmol of inorganic P per min from sodium phytate at pH 5–5 and 37°C. One amylase unit is equal to the amount of enzyme required to break down 1 M glucosidic linkage per min at a temperature of 37°C and a pH of 7.0. Units of xylanase, glucanase and pectinase are measured by their ability to hydrolyze wheat arabinoxylan, barley glucan or pectin, respectively, at pH 5.0 at 50°C to yield 1 mol of total reducing sugar equivalents (as xylose, glucose or galacturonic acid) per min. At 40°C and a pH of 3.5, one unit of protease activity was defined as the amount of enzyme required to release 1 μg of tyrosine per min from 20 mg/mL of casein.

^b^SN diet, standard nutrient diets (adequate in all nutrients); LN, low‐nutrient diet (reduced 100 kcal/kg of metabolizable energy and reduced 5% of crude protein and limiting amino acids, including lysine, methionine + cysteine, and threonine, in a calculated amount relative to the SN diet); +0, no enzymes added; +PHY, phytase added; +PHY+FEC, phytase and feed enzyme complex added.

### 2.4 Growth performance measurements

Body weight (BW) and feed intake were assessed at the conclusion of the starter, grower and finisher phases. The average daily feed intake (ADFI), average daily gain (ADG) and feed conversion ratio (FCR; ADFI/ADG) were calculated for each feeding phase and throughout the whole trial (0–42 days). Additionally, daily records of mortality and health status were kept to calibrate the growth performance. The calculation of the uniformity rate involved the utilization of the coefficient of variation of the BW of each individual at 42 days of age, as expressed in the subsequent formula:

$$\mathrm{Uniformity}\;\mathrm{rate}=100-\left(\left[\mathrm{standard}\;\mathrm{deviation}/\mathrm{average}\;\mathrm{BW}\right]\times 100\right).$$

From days 0 to 42, the European production efficiency index (EPEI) of each experimental unit was calculated using the formula:

$$\mathrm{EPEI}=\mathrm{livability}\left(\%\right)\times \mathrm{BW}\left(\mathrm{kg}\right)\times 100/\mathrm{age}\left(d\right)\times \mathrm{FCR}$$

### 2.5 Sample collection and procedures

On day 42, 2 birds/pen were randomly selected and euthanized by cervical dislocation. For morphological analysis, approximately 2‐cm fragments were taken from the middle part of the duodenum and jejunum and submerged in approximately 25 mL of 10% neutral‐buffered formalin after being rinsed with distilled water. Furthermore, the tibiae located on both the right and left sides of each avian specimen were meticulously dissected and thoroughly purged of any attached tissues. Measurements were conducted on the weight, length and diameter of the right tibia. The left tibia was preserved at −20°C and subsequently analysed for its ash content, as well as the levels of calcium (Ca) and phosphorus (P).

On day 42, a total of 72 chickens (2 chickens/pen) were selected to measure in vivo nutrient digestibility. The acid‐insoluble ash (AIA) marker method was used to examine nutrient digestibility during the course of a 4‐day experiment. On day 42, 4 days prior to excreta collection, Celite (Celite 545, Merck KGaA) was included in the diet at a rate of 10 g/kg to serve as an extra source of AIA. The luminal contents of the ileum (the segment of the gastrointestinal tract located between Meckel's diverticulum and the ileocecal junction) were aseptically collected in sterile plastic bags. A sample of ileal digesta from two avian specimens housed in the same replicate pen was combined and subsequently subjected to freezing at −20°C for the purpose of conducting a comprehensive analysis of its digestibility.

### 2.6 Tibia bone measurements

The length and weight of the tibia were measured using the right tibial bones. A digital calliper accurate to 0.01 mm was used to measure tibial diameters at the broadest and narrowest points, and the mean of these measurements was used for statistical analysis. The left tibia samples were crushed and defatted for 24 h using the Soxhlet apparatus and petroleum ether before being oven‐dried at 100°C for 24 h. The content of bone ash in dried samples was determined by burning them in a muffle furnace at 600°C for 24 h (fat‐free, dry weight basis). The P and Ca concentrations of the tibia samples were determined using conventional protocols after the ash was solubilized using a nitric and perchloric acid combination (Association of Official Analytical Chemists [AOAC], 2005; method 964.06 for P and method 968.08 for Ca).

### 2.7 Nutrient digestibility

The samples of feed and ileal digesta were subjected to chemical analysis after undergoing a drying process in an oven at 65°C for a duration of 24 h. Subsequently, the samples were pulverized into a fine powder. The feed and ileal digesta samples were dried in an oven at 65°C for 24 h and then crushed into a fine powder for chemical analysis. Next, the samples were analysed using the AOAC (2005) to determine the contents of dry matter (DM; method 930.15), crude fat (method 920.39), CP (*N* × 6.25; method 984.13) and crude ash (method 942.05). Organic matter (OM) was calculated through subtraction of ash percentage from 100%. An automated adiabatic oxygen bomb calorimeter (Parr Instrument Company) was also used to measure the gross energy of the samples. The content of AIA in the feed and ileal samples was quantified, according to McCarthy et al. (1974). Apparent ileal digestibility coefficients (AIDCs) of nutrients were calculated using the following equation:

$$\mathrm{AIDC}=\left[{\left(\mathrm{Nutr}/\mathrm{AIA}\right)}_{\mathrm{feed}}-{\left(\mathrm{Nutr}/\mathrm{AIA}\right)}_{\mathrm{id}}\right)]/{\left(\mathrm{Nutr}/\mathrm{AIA}\right)}_{\mathrm{feed}}$$

where (Nutr/AIA)_feed_ represents the ratio of nutrient to AIA in the feed, and (Nutr/AIA)_id_) reflects the ratio of nutrient to AIA in the ileal digesta.

For the measurement of apparent ME adjusted for nitrogen balance (AMEn), excreta samples were collected (*n* = 6 per treatment). The following equation was used to calculate the AMEn value (Majdolhosseini et al., 2019):

$$\begin{array}{ccc} & & \mathrm{AMEn}\;\left(\mathrm{kcal}/\mathrm{kg}\;\mathrm{of}\;\mathrm{feed}\right)=GE_{\mathrm{feed}}\\ & & \;-\;[\left(GE_{\mathrm{excreta}}\times \mathrm{IF}\right)+8.22\times \left(N_{\mathrm{feed}}-N_{\mathrm{excreta}}\times \mathrm{IF}\right)] \end{array}$$

where GE_feed_ is gross energy content in feed (kcal/kg) and GE_excreta_ is gross energy content in excreta (kcal/kg), IF is the indigestibility factor (AIA_feed_/AIA_excreta_), N _feed_ is nitrogen content in feed (%), N_excreta_ is nitrogen content in excreta (%) and 8.22 is the energy equivalent (kcal/g) of uric acid.

### 2.8 Gut morphology

A 5‐μm cross‐section of each intestinal tissue segment was cut with a microtome, mounted on a glass slide, stained with haematoxylin–eosin and seen under a light microscope (Olympus, CX31) for analysis. Three cross‐sections of each intestinal sample were taken, with 10 measurements per each variable. The morphometric parameters, including villus height (VH), villus width (VW) and crypt depth (CD), were measured using image‐analysis software (QWin Plus v. 3.1.0; Leica Cambridge Ltd.). The measurement of VH was conducted from the base to the apex of the villi, whereas VW was determined by measuring the width of a selected villus at three different locations (i.e. top, middle and bottom) and computing the mean of the three measurements. Additionally, CD was measured from the junction of the crypt‐villus to the crypt base. The VH/CD ratio was determined by utilizing data from both the VH and the CD. Furthermore, the surface area of the villus (VSA) was calculated using the subsequent formula: 2*π* × (VW/2) × VH.

### 2.9 Statistical analysis

The data were examined using a 2 × 3 factorial design with a general linear model (GLM). The first factor was the diet (SN vs. LN), and the second factor was the enzyme treatment, which included control (no enzymes), PHY and PHY + FEC. A comparative analysis among means was conducted using the following orthogonal contrasts: control versus PHY, control versus PHY + FEC and PHY versus PHY + FEC. The normality and homogeneity of variances were assessed using the Shapiro–Wilk and Levene tests, respectively. Means were separated using Tukey's post hoc analysis, and differences were considered statistically significant at *p* < 0.05. Results are expressed as means with standard errors of the mean. All statistical analyses were performed using the SAS Statistical Package (version 9.0, SAS Institute).

## 3 RESULTS

### 3.1 Productive performance

The effect of diet quality and supplemental PHY and FEC on growth performance in broiler chickens is shown in Table 3. During the entire growing period (0–42 days), the BW, ADG, ADFI, uniformity rate and EPEI in the birds fed with the LN diet were lower (*p* < 0.001) than those in birds fed with the SN diet. The FCR in the LN group was also higher (*p* < 0.05) than that in the SN group. Moreover, the lower quality of the LN diet tended to increase (*p* = 0.065) the mortality rate. The main effect of enzyme supplementation led to higher (*p* < 0.05) final BW, overall ADG and EPEI in the PHY and PHY + FEC treatments compared to the control treatment, with the highest values observed in the PHY + FEC treatment. The PHY + FEC treatment also decreased (*p* < 0.05) the FCR during the overall experimental period. In addition, there was a tendency towards a higher uniformity rate (*p* = 0.083) in the PHY + FEC group. There was a diet × enzyme factorial effect on final BW, ADG and EPEI, with the enzyme treatments, particularly PHY + FEC, showing a more pronounced impact on the LN‐fed broilers.

**TABLE 3:** Effect of phytase (PHY) and a feed enzyme complex (FEC) supplementation to lower nutrient diets on growth performance^1^ of broiler chickens at 42 days of age.

Treatments^2^		BW (g/bird)	ADG (g/d bird)	ADFI (g/d bird)		Uniformity (%)	Mortality (%)	EPEI^2^
Diet	Enzyme				FCR			
SN	+0	2585^a^	60.52^a^	112.4	1.86	90.18	3.57	319.9^a^
	+PHY	2599^a^	60.87^a^	112.2	1.85	91.24	4.76	319.4^a^
	+PHY+FEC	2613^a^	61.18^a^	112.2	1.83	92.58	2.98	329.1^a^
LN	+0	2427^c^	56.76^c^	109.6	1.93	86.86	7.14	278.0^c^
	+PHY	2521^b^	58.99^b^	111.4	1.89	89.35	4.17	304.5^b^
	+PHY+FEC	2585^a^	60.52^a^	111.3	1.85	90.14	5.36	316.9^ab^
SEM		16.6	0.396	0.78	0.018	1.235	1.144	5.01
Main effects								
Diet types^3^								
SN		2599^a^	60.85^a^	112.3^a^	1.85^b^	91.33^a^	3.77	322.8^a^
LN		2511^b^	58.76^b^	110.8^b^	1.89^a^	88.78^b^	5.56	299.8^b^
SEM		9.6	0.229	0.45	0.010	0.713	0.661	2.89
Enzyme								
+0		2506^c^	58.64^c^	111.0	1.89^a^	88.52	5.36	298.9^c^
+PHY		2560^b^	59.93^b^	111.8	1.87^ab^	90.30	4.46	312.0^b^
+PHY+FEC		2599^a^	60.85^a^	111.8	1.84^b^	91.36	4.17	323.0^a^
SEM		11.7	0.280	0.55	0.013	0.873	0.809	3.54
Probability								
Diet		<0.001	<0.001	0.023	0.011	0.017	0.066	<0.001
Enzyme		<0.001	<0.001	0.466	0.015	0.083	0.563	<0.001
Diet × enzyme		0.001	0.002	0.383	0.212	0.843	0.189	0.010
Contrast comparisons								
+0 vs. +PHY		0.003	0.002	0.271	0.149	0.161	0.442	0.014
+0 vs. +PHY+FEC		<0.001	<0.001	0.306	0.004	0.029	0.307	<0.001
+PHY vs. +PHY+FEC		0.025	0.025	0.936	0.115	0.395	0.797	0.035

*Note*: Means represent 6 pens of 25 chicks each. Means within the same column not sharing a common letter are significantly different (*p* < 0.05).

Abbreviation: SEM, standard errors of the mean.

^1^BW, body weight; ADG, average daily gain; ADFI, average daily feed intake; FCR, feed conversion ratio; EPEI, European production efficiency index = livability (%) × live weight (kg) × 100/age (d) × FCR.

^2^SN diet, standard nutrient diets (adequate in all nutrients); LN, low‐nutrient diet (reduced 100 kcal/kg of metabolizable energy and reduced 5% of crude protein and limiting amino acids, including lysine, methionine + cysteine and threonine, in a calculated amount relative to the SN diet); +0, no enzymes added; +PHY, phytase added; +PHY+FEC, phytase and feed enzyme complex added.

### 3.2 Ileal nutrient digestibility

The effect of diet quality and supplemental PHY and FEC on the AID of nutrients in broiler chickens is shown in Table 4. Two‐way interaction effects of diet × enzyme were not significant on ileal nutrient digestibility or AMEn. Although the AID of DM, OM, CP, crude fat and energy were not affected by the diet quality (*p* > 0.05), the AMEn of the LN group was lower (*p* < 0.05) than that of the SN group. The PHY and PHY + FEC treatments increased (*p* < 0.05) the AID of CP compared to the control treatment. The PHY + FEC treatment also increased (*p* < 0.05) the AMEn and AID of DM, OM and energy but had no effect on the AID of crude fat (*p* > 0.05).

**TABLE 4:** Effect of phytase (PHY) and a feed enzyme complex (FEC) supplementation to lower nutrient diets on effect of enzyme supplementation to the low‐nutrient diets on dietary nutrient digestibility (%) and nitrogen‐corrected apparent metabolizable energy (AMEn) value (kcal/kg) in broiler chickens.

Treatments^1^							
Diet	Enzyme	Dry matter	Crude protein	Crude fat	Organic matter	Energy	AMEn
SN	+0	75.7	68.7	82.2	79.6	73.1	3180
	+PHY	76.5	70.8	83.4	80.8	74.8	3194
	+PHY+FEC	78.2	71.9	84.1	81.4	76.3	3212
LN	+0	75.1	69.9	82.9	80.2	73.6	3068
	+PHY	76.7	71.4	83.5	81.6	75.1	3096
	+PHY+FEC	77.6	72.2	85.0	81.3	76.6	3119
SEM		0.90	0.77	1.00	0.58	0.81	10.9
Main effects							
Diet types							
SN		76.8	70.5	83.2	80.6	74.7	3196^a^
LN		76.5	71.2	83.8	81.0	75.1	3094^b^
SEM		0.52	0.45	0.57	0.34	0.47	6.3
Enzyme							
+0		75.4^b^	69.3^b^	82.5	79.9^b^	73.3^b^	3123^b^
+PHY		76.6^ab^	71.1^a^	83.5	81.2^a^	74.9^ab^	3145^ab^
+PHY+FEC		77.9^a^	72.0^a^	84.6	81.3^a^	76.5^a^	3166^a^
SEM		0.64	0.54	0.70	0.41	0.57	7.7
Probability							
Diet		0.664	0.287	0.491	0.386	0.575	<0.001
Enzyme		0.036	0.004	0.138	0.036	0.002	0.003
Diet × enzyme		0.860	0.823	0.915	0.799	0.986	0.661
Contrast comparisons							
+0 vs. +PHY		0.184	0.024	0.365	0.034	0.063	0.066
+0 vs. +PHY+FEC		0.010	0.001	0.048	0.020	<0.001	0.001
+PHY vs. +PHY+FEC		0.179	0.247	0.261	0.811	0.061	0.068

*Note*: Means represent 6 pens of 25 chicks each. Means within the same column not sharing a common letter are significantly different (*p* < 0.05).

Abbreviation: SEM, standard errors of the mean.

^1^SN diet, standard nutrient diets (adequate in all nutrients); LN, low‐nutrient diet (reduced 100 kcal/kg of metabolizable energy and reduced 5% of crude protein and limiting amino acids, including lysine, methionine + cysteine and threonine, in a calculated amount relative to the SN diet); +0, no enzymes added; +PHY, phytase added; +PHY+FEC, phytase and feed enzyme complex added.

### 3.3 Bone characteristics

Data on the morphological characteristics and mineral contents of the tibia are shown in Table 5. Tibia length and diameter, as well as Ca concentration in the tibia, were lower in the LN group than in the SN group (*p* < 0.05). The tibia weight and tibia ash content also tended to be lower (*p* = 0.062 and 0.052, respectively) in birds fed the LN diet. Regarding the main effect of enzyme supplementation, the inclusion of PHY alone or in combination with FEC resulted in an increase (*p* < 0.05) in tibia diameter, ash content and P concentration. A diet × enzyme interaction was observed for tibia ash concentration (*p* < 0.05), suggesting that the response to PHY + FEC treatment was more pronounced in the LN group compared to the SN group.

**TABLE 5:** Effect of phytase (PHY) and a feed enzyme complex (FEC) supplementation to the low‐nutrient diet on tibia bone characteristics in broiler chickens.

Treatments^1^		Tibia weight, g	Tibia length, cm	Tibia diameter, mm	Tibia ash, %	Tibia calcium^2^, g/kg	Tibia phosphorous^2^, g/kg
Diet	Enzyme						
SN	+0	10.19	15.49	8.03	52.80^ab^	170.0	90.27
	+PHY	10.28	16.19	8.42	54.43^a^	173.8	92.78
	+PHY+FEC	10.30	16.53	8.39	53.62^ab^	173.5	92.86
LN	+0	9.66	14.50	7.35	50.73^c^	162.5	88.46
	+PHY	10.10	15.22	7.96	52.30^bc^	166.5	91.97
	+PHY+FEC	10.03	15.10	8.17	54.48^a^	169.5	93.23
SEM		0.209	0.409	0.211	0.666	2.65	1.205
Main effects							
Diet types							
SN		10.26	16.07^a^	8.28^a^	53.62	172.4^a^	91.97
LN		9.93	14.94^b^	7.83^b^	52.50	166.2^b^	91.22
SEM		0.121	0.236	0.122	0.385	1.53	0.696
Enzyme							
+0		9.93	14.99	7.69^b^	51.76^b^	166.3	89.37^b^
+PHY		10.19	15.71	8.19^a^	53.36^a^	170.2	92.37^a^
+PHY+FEC		10.16	15.81	8.28^a^	54.05^a^	171.5	93.05^a^
SEM		0.130	0.307	0.155	0.471	2.08	0.889
Probability							
Diet		0.063	0.002	0.013	0.052	0.007	0.450
Enzyme		0.388	0.109	0.018	0.006	0.139	0.011
Diet × enzyme		0.688	0.818	0.566	0.049	0.764	0.666
Contrast comparisons							
+0 vs. +PHY		0.091	0.212	0.024	0.023	0.149	0.018
+0 vs. +PHY+FEC		0.054	0.266	0.008	0.002	0.058	0.005
+PHY vs. +PHY+FEC		0.795	0.887	0.655	0.311	0.623	0.579

*Note*: Means represent 6 pens of 25 chicks each. Means within the same column not sharing a common letter are significantly different (*p* < 0.05).

Abbreviation: SEM, standard errors of the mean.

^1^SN diet, standard nutrient diets (adequate in all nutrients); LN, low‐nutrient diet (reduced 100 kcal/kg of metabolizable energy and reduced 5% of crude protein and limiting amino acids, including lysine, methionine + cysteine and threonine, in a calculated amount relative to the SN diet); +0, no enzymes added; +PHY, phytase added; +PHY+FEC, phytase and feed enzyme complex added.

^2^ Based on fat‐free dry tibia weight.

### 3.4 Gut morphology

Morphological features in the duodenum and jejunum of broiler chickens given various experimental diets are shown in Table 6. The duodenal VH tended (*p* = 0.096) to be lower in the LN group compared to the SN group, but none of the other morphological parameters of the duodenum were affected by the dietary nutrient density. In the jejunum, VH and VSA were lower (*p* < 0.05) in birds fed the LN diet as compared to those fed the SN diet. The inclusion of FEC on top of PHY increased (*p* < 0.05) VH/CD ratio and also tended to increase VH (*p* = 0.081) and VSA (*p* = 0.091) in the duodenum. The VH, VH/CD and VSA in the jejunum in PHY and PHY + FEC treatments were also greater (*p* < 0.05) than those in the control treatment, where the PHY + FEC treatment had the highest values. In addition, the jejunal VW in birds fed with the PHY + FEC diet was higher (*p* < 0.05) than that in birds fed with the control diet. The jejunal VH and VSA were also influenced by diet × enzyme interaction, so that PHY + FEC treatment was more effective in enhancing these parameters in birds fed the LN diet compared to those fed the SN diet (*p* < 0.05).

**TABLE 6:** Effect of phytase (PHY) and a feed enzyme complex (FEC) supplementation to the low‐nutrient diet on intestinal morphological variables^1^ in broiler chickens.

Treatments^2^		Duodenum					Jejunum				
Diet	Enzyme	VH (μm)	VW (μm)	CD (μm)	VH/CD	VSA (mm^2^)	VH (μm)	VW (μm)	CD (μm)	VH/CD	VSA (mm^2^)
SN	+0	1839	173.5	250.5	7.33	1.003	1495^b^	152.9	247.0	6.08	0.720^bc^
	+PHY	1882	176.1	246.6	7.75	1.048	1590^a^	159.3	235.0	6.83	0.795^ab^
	+PHY+FEC	1913	180.5	241.2	8.06	1.090	1603^a^	162.0	234.8	6.96	0.817^a^
LN	+0	1705	169.8	257.6	6.62	0.906	1341^c^	147.2	256.1	5.26	0.620^d^
	+PHY	1775	171.0	250.9	7.13	0.950	1424^bc^	154.4	242.9	5.91	0.687^cd^
	+PHY+FEC	1906	179.7	240.9	7.98	1.076	1596^a^	166.6	222.8	7.21	0.836^a^
SEM		61.6	6.78	9.83	0.392	0.0584	32.8	4.92	9.64	0.315	0.0274
Main effects											
Diet types											
SN		1878	176.7	246.1	7.71	1.047	1563^a^	158.1	238.9	6.62	0.777^a^
LN		1795	173.5	249.8	7.24	0.977	1454^b^	156.1	240.6	6.12	0.715^b^
SEM		34.2	3.91	5.67	0.224	0.0330	18.9	2.84	5.56	0.182	0.0158
Enzyme											
+0		1772	171.6	254.1	6.98^b^	0.955	1418^c^	150.1^b^	251.6	5.67^c^	0.670^c^
+PHY		1829	173.6	248.8	7.44^ab^	0.999	1507^b^	156.8^ab^	238.9	6.37^b^	0.741^b^
+PHY + FEC		1909	180.1	241.0	8.02^a^	1.083	1600^a^	164.3^a^	228.8	7.08^a^	0.826^a^
SEM		41.9	4.80	6.96	0.275	0.0404	21.2	3.48	6.81	0.223	0.0194
Probability											
Diet		0.096	0.567	0.649	0.147	0.150	<0.001	0.625	0.835	0.062	0.009
Enzyme		0.081	0.440	0.421	0.041	0.091	<0.001	0.025	0.077	<0.001	<0.001
Diet × enzyme		0.539	0.948	0.932	0.681	0.709	0.038	0.517	0.481	0.140	0.049
Contrast comparisons											
+0 vs. +PHY		0.342	0.781	0.595	0.249	0.440	0.011	0.179	0.199	0.033	0.013
+0 vs. +PHY+FEC		0.027	0.225	0.195	0.012	0.032	<0.001	0.007	0.025	<0.001	<0.001
+PHY vs. +PHY+FEC		0.182	0.346	0.436	0.147	0.154	0.008	0.140	0.303	0.031	0.005

*Note*: Means represent 6 pens of 25 chicks each. Means within the same column not sharing a common letter are significantly different (*p* < 0.05).

Abbreviation: SEM, standard errors of the mean.

^1^VH, villus height; VW, villus width; CD, crypt depth, VSA, villus surface area (mm^2^) = 2π × (VW/2) × VH.

^2^SN diet, standard nutrient diets (adequate in all nutrients); LN, low‐nutrient diet (reduced 100 kcal/kg of metabolizable energy and reduced 5% of crude protein and limiting amino acids, including lysine, methionine + cysteine and threonine, in a calculated amount relative to the SN diet); +0, no enzymes added; +PHY, phytase added; +PHY+FEC, phytase and feed enzyme complex added.

**FIGURE 1:**
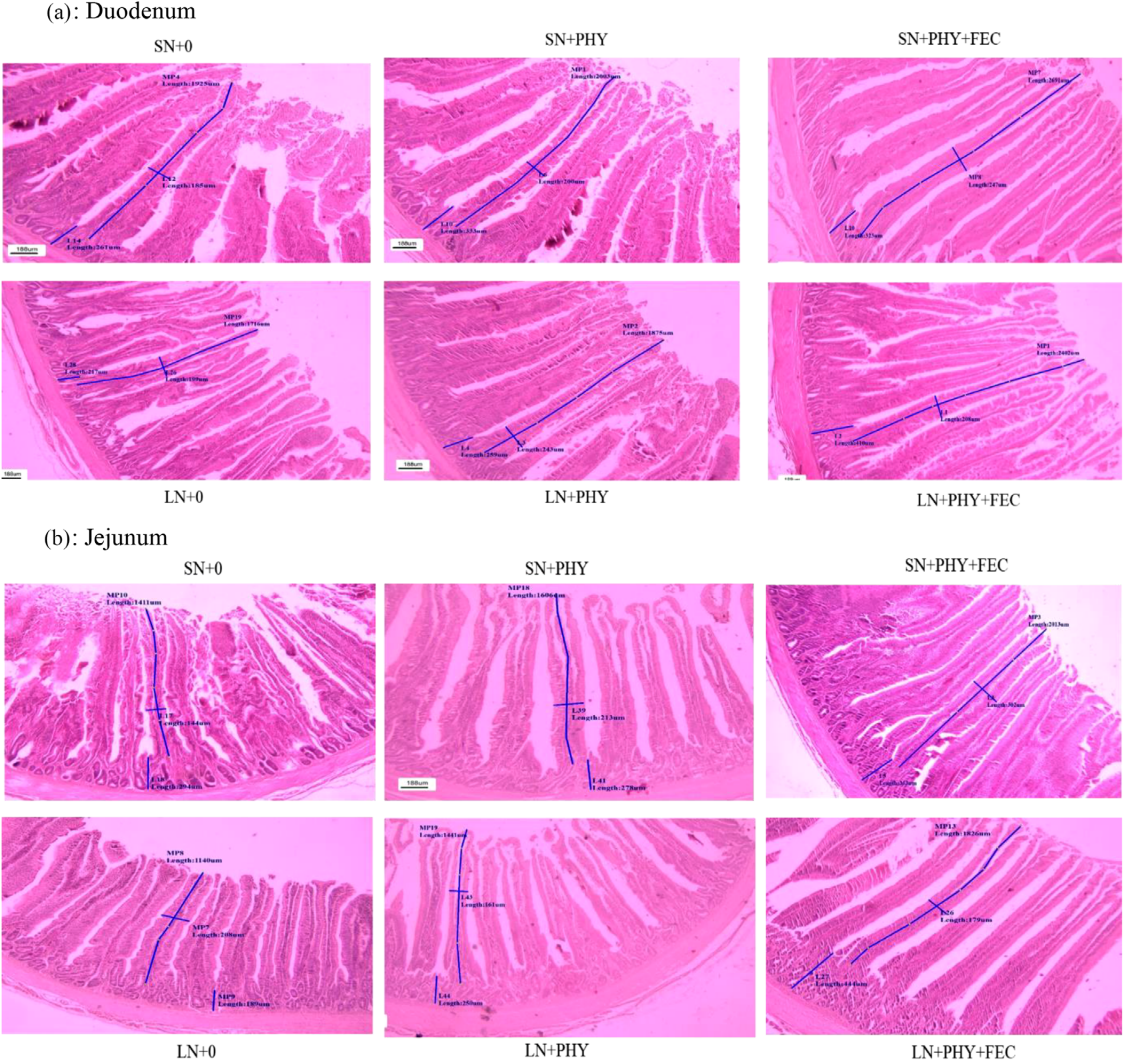
The histological features of the (A) duodenum and (B) jejunum mucosa (based on haematoxylin and eosin staining) in 42‐day‐old broiler chickens (replicate 3 from all treatments). For histological observation, images at a lower magnification (100×) are provided. SN diet, standard nutrient diets (adequate in all nutrients); LN, low‐nutrient diet; +0, no enzymes added; +PHY, phytase added; +PHY+FEC, phytase and feed enzyme complex added.

Figure 1 depicts the histological characteristics of the mucosa in the duodenum and jejunum. There is concordance between the revealed morphological characteristics and the numerical statistics. Figure 1 demonstrates that the SN group had more uniform, longer and closely spaced intestinal villi compared to the LN grout, especially in the jejunum mucosa. The groups that received enzyme supplementation, particularly those that received PHY + FEC supplementation, displayed improved villi structure, characterized by longer villi and a more uniform arrangement.

## 4 DISCUSSION

In the present study, reductions in dietary AMEn, CP and LAA decreased feed intake from 0 to 42 days, with the greatest reduction occurring during the finisher period. As a result of the decrease in feed intake, the LN group experienced an overall decline in their ADG, FCR, uniformity rate and EPEI. This impairment in growth performance could be attributed to the dietary energy and CP–LAA dilution applied. Similarly, growth performance parameters were reduced as a consequence of the lower nutrient density of the diet (Boontiam et al., 2017; Chen et al., 2013; Jlali et al., 2020).

As shown by the interaction effect, including PHY alone or in combination with FEC in the diet increased final BW, overall ADG and EPEI, with the effect being more pronounced in the LN diet–fed group. However, despite this improvement in growth performance with the addition of PHY supplement to the LN diet, the overall growth rate of these birds remained lower than the value of chickens fed the SN diet. This indicates that, although PHY supplementation at 1000 FTU/kg of feed improved energy and nutrient utilization (as shown in the current study), these chickens still suffered from a deficiency of energy, CP and LAA when compared to chickens in the control group. In another study on broilers, Dersjant‐Li et al. (2018) discovered that the use of a Buttiauxella PHY at 500 and 1000 FTU/kg of feed could compensate for a reduction in nutrients (−1.46 g/kg available P, −0.2 g/kg dig AA, −67 kcal/kg ME, −0.3 g/kg Na and −2.3 g/kg Ca) and maintain growth performance over a 42‐day period compared with diets based on breeder recommendations. Attia et al. (2021) also discovered that although supplementing low‐density diets (−1% CP and −100 kcal/kg ME) with either 500 FTU/kg of an *E. coli* PHY or 500 U/kg of an *Aspergillus niger* PHY improved the growth performance of coloured broilers of the Sasso strain during 1–64 days of age, their final BW and feed intake were still lower than the non‐supplemented control birds. Several factors, including the severity and duration of dietary nutrient reduction, the composition of the bird's base diet, the strain and the bird's physiological state, are likely responsible for this disparity in results.

According to the findings of this study, the addition of FEC to the LN diet supplemented with PHY could exert an extra beneficial effect on growth rate and performance index, allowing for a reduction of ME (−100 kcal/kg) and CP–LAA (up to 5% of the recommended level) in the Ca and P adequate diets for broiler chickens. One possible explanation is that the concurrent use of FEC and PHY led to a greater hydrolysis of NSP (cell wall components), allowing the PHY enzyme to more easily access the phytate contained within the plant cells (Cowieson et al., 2006). As indicated by the data presented in Table 2, the enzyme composition of the multi‐enzyme supplement primarily consisted of carbohydrase enzymes, with a specific emphasis on NSP‐decomposing enzymes including xylanase, β‐glucanase and pectinase. This observation suggests that the beneficial impact of this supplement on growth performance measures may primarily be attributed to the presence of enzymes in the FEC preparation. In addition, the presence of protease enzyme in FEC (3000 PCT/kg diet), in addition to NSP degrading enzymes, can assist in the improvement of protein digestion in the diet, which ultimately results in more amino acid release. The positive impact of PHY and FEC enzymes found in the current research is consistent with findings from previous studies conducted on broiler chickens given corn–soya bean meal–based diets (Gallardo et al., 2017; Józefiak et al., 2010; Lu et al., 2013). In a recent study, Bavaresco et al. (2020) reported that feeding broiler chickens a diet containing 500 FTU/kg of hybrid PHY along with 560 TXU/kg of xylanase and 250 TGU/kg of glucanase helped them maintain BW and daily weight gain despite a reduction in dietary energy (−100 kcal/kg), Ca (−0.16%) and available P (−0.15%). The positive effect of PHY + FEC treatment on the performance of broiler chickens fed with the LN diets can be explained by the improvements in ileal nutrient digestibility (DE +3.3%; CP +3.9%; OM +1.8%; energy +4.4%; AMEn +43 kcal/kg) and morphological characteristics of the intestinal mucosa observed in the current study.

Similar to the results of the current study, Upadhaya et al. (2019) found no effect of different dietary energy and protein concentrations (100% ME and CP, 98.8% ME and CP and 97.6% ME and CP) on nutrient digestibility in broiler chickens. In addition, Majdolhosseini et al. (2019) revealed that digestibility coefficients of DM, CP and crude fat were not influenced by reducing the dietary ME level (−100 kcal/kg) in broiler chicks. This study showed that on day 46, the IDC for CP was improved by the addition of PHY to the diet. Similarly, Zarghi et al. (2022) reported that the dietary inclusion of PHY (500 FTU/kg) improved nutrient digestibility in broiler chickens. The favourable effects of PHY on nutrient digestibility may be connected to phytate breakdown and the supply of nutrients other than P, which would enable the host to use additional nutrients (Gallardo et al., 2020). In addition, Liu and Ru (2010) found that supplementing the feed of broiler chickens with 500 FTU/kg of *E. coli*–derived PHY reduced the endogenous amino acid losses in the intestine, which may account for the higher ileal CP digestibility observed in this study. In the current research, dietary supplementation with FEC on top of PHY improved the IDCs of DM, OM and energy, as well as AMEn. Our findings support the findings of recent meta‐analysis studies (Kiarie et al., 2021; Llamas‐Moya et al., 2021), which found that the presence of NSP in corn–soya bean meal–based diets reduces nutrient digestibility and that the addition of a carbohydrase such as β‐mannanase and α‐galactosidase to animal diets increases overall nutrient digestibility. The presence of mannans, galactomannans and pectins as primary components of NSP in soya bean meal results in an increase in the viscosity of digesta, which may have a detrimental impact on nutritional digestion and absorption in the gastrointestinal system (Amer et al., 2020; Mohammadigheisar et al., 2021). It is possible that FEC enzyme preparation used in this study, which contains different carbohydrase enzymes, could hydrolyze NSP compounds, thereby releasing oligosaccharides that function as prebiotics and promote the survival and growth of lactic acid bacteria in the gut. This, in turn, lowers the pH of the gastrointestinal tract, which generally leads to better absorption of nutrients in animals (Raza et al., 2019; Abbasi Arabshahi et al., 2021).

Bone health in poultry can be assessed with the evaluation of tibial morphological measurements like length, weight, ash and mineral content (Onyango et al., 2003; Venäläinen et al., 2006). Among the assessed bone quality parameters, the tibia weight, diameter and ash content were negatively influenced by the LN diets (−100 kcal/kg ME and −5% CP–LAA), indicating that a lower quantity of bone components was available in the LN group. The decrease in feed intake and, consequently, mineral intake caused by feeding the LN diets in the current study may be the main factor negatively affecting bone quality. The current findings also suggest that, regardless of lower dietary ME and CP–LAA levels, PHY supplementation in the diet has a positive effect on bone mineralization by increasing tibial ash and P content. This enhancement in tibia mineralization reported in broilers given a diet supplemented with PHY is comparable with that found in previously published research on broilers (Akter et al., 2016; Jing et al., 2021; Mohammadi Ziarat et al., 2020). In the present work, PHY was employed to dephosphorylate myo‐inositol hexaphosphate, which likely enhanced bone mineralization by improving the bioavailability of ash, P and Ca. In the current investigation, there was a two‐way interaction between diet and enzyme for tibia ash content, indicating that the beneficial effect of PHY + FEC was more pronounced in the LN group. The efficiency of FEC in enhancing bone strength may be related to the utilization of minerals, particularly P and Ca, because optimizing bone mineralization is a significant factor affecting bone strength (Rath et al., 2000). Similarly, Wang et al. (2019) indicated that extra‐dosing PHY or combining PHY with FEC could improve phytate‐P release and bone mineralization parameters in broiler chickens. It seems that the inclusion of FEC breaks down NSP clustered with phytic acid and increases the accessibility of PHY to the substrate.

Morphological properties of the small intestine mucosal surface provide insight into the absorptive process and developmental progression of the small intestine (Yang et al., 2007). An increase in the VH/CD ratio is associated with increased digestion and absorption in the gut because longer villi and shorter crypts give greater surface area (Jelveh et al., 2022). Our findings revealed that while the reduction of ME and CP–LAA levels did not have a detrimental effect on the duodenal morphology, it did lead to a decrease in the VH and VSA in the jejunum. Previous research found no significant differences in the intestinal morphological features of broiler chickens when dietary ME was reduced by 100 kcal/kg (Wickramasuriya et al., 2019) or 150 kcal/kg (Attia et al., 2021). In this study, it appears that the detrimental effect of dietary nutrient restriction on the morphological structure of the jejunum is related more to the decrease in dietary protein than to the decrease in dietary ME. Similarly, a recent study has shown that feeding low‐CP diets (95% of the Ross 308 recommendations for CP) adversely affected the jejunal morphology by decreasing VH, VW and VSA in heat‐stressed broilers (Ghasemi et al., 2021). It has been reported that glycine and serine, together with cysteine and proline, are fundamental components of the unstirred water layer of the mucosa, which, accompanied by threonine, plays a significant role in villus production (Liu et al., 2017). Consequently, the decreased supply of these amino acids for small intestinal mucosal‐cell proliferation may have contributed to the impaired intestinal morphology found in broiler chickens fed the LN diets compared to those fed the SN diets in this study.

The findings of this study are comparable with those of other researchers, who have found that PHY supplementation improves the morphometric aspects of the small intestine mucosa in broiler chickens (Smulikowska et al., 2010) and Japanese quails (Sajadi Hezaveh et al., 2020). It has been observed that supplementing with PHY may inhibit the development of intestinal pathogenic bacteria by limiting the amount of accessible substrates for their metabolization (Borda‐Molina et al., 2019; Nari et al., 2020). Because of this, the intestinal mucosa sustains less inflammatory damage, which benefits the VH and secretion processes (Cook & Bird, 1973), leading to enhanced nutritional digestion and absorption through the mucosa of the intestinal villi. As presented in Table 6, jejunal VH and VSA were influenced by diet × enzyme interaction, so that the jejunal morphology of chickens fed LN diets was more positively affected by dietary supplementation with PHY and FEC than that of chickens fed SN diets. Similarly, significant improvements were observed in the intestinal morphological indicators of broiler chickens as a result of glucanase and/or mannanase supplementation (Karimi and Zhandi, 2014; Wang et al., 2005). By contrast, the addition of xylanase and β‐glucanase to the diets had no impact on the intestinal morphology of broiler chickens (Kalmendal and Tauson, 2012). The positive change in intestinal morphology after enzyme supplementation might partially account for the observed improvement in growth performance indices in the current investigation. The mechanism of action by which multi‐enzyme improves intestinal histomorphology is not well understood. The supplementation of carbohydrase, especially β‐mannanase in corn–soya bean meal–based diets might alleviate the rise in digesta viscosity due to the presence of β‐mannan derived from soya bean meal, which negatively affects the intestinal mucosa by exposing the mucosal surface to the viscous substances (Kim et al., 2021).

## 5 CONCLUSIONS

In conclusion, an LN diet (low AMEn, CP and LAAs) had a negative impact on growth performance, morphological indices, ash and Ca levels in the tibia, as well as jejunal morphological parameters, in broiler chickens. The inclusion of PHY, particularly when combined with multi‐enzyme supplementation, had a positive impact on growth performance in diets with restricted nutrient content. The simultaneous use of PHY and FEC enzymes also improved intestinal morphology in broilers on an LN diet compared to broilers on a standard diet. However, the benefits of these enzymes were independent of dietary nutrient density in terms of nutrient digestibility and tibia quality metrics.

## AUTHOR CONTRIBUTIONS

*Conceptualization; investigation; data curation; software*: Mostafa Ahmadi. *Project administration; supervision; validation; writing – original draft; writing – review and editing*: Hossein Ali Ghasemi. *Formal analysis; resources; writing – review and editing*: Iman Hajkhodadadi. *Methodology; funding acquisition; writing – review and editing*: Farhad Khaligh. All authors have read and confirmed the final version of the manuscript.

## CONFLICT OF INTEREST STATEMENT

The authors declare that the present study was carried out without any commercial or financial affiliation that could be perceived as a possible source of conflict of interest.

## ETHICS STATEMENT

The authors attest that they have obtained approval from the Arak University Institutional Animal Care and Use Committee (contract number 1400/1911) and have followed the journal's ethical policies as outlined on the journal's author guidelines page. The authors have also complied with all regulations regarding the protection of animals used for scientific purposes.

### PEER REVIEW

The peer review history for this article is available at https://publons.com/publon/10.1002/vms3.1344.

## Data Availability

Data are available on request from the authors.
